# A rare intestinal mucormycosis caused by *Lichtheimia ramosa* in a patient with diabetes: a case report

**DOI:** 10.3389/fmed.2024.1435239

**Published:** 2024-10-16

**Authors:** Qinqin Liu, Ping Chen, Li Xin, Jiahao Zhang, Meijie Jiang

**Affiliations:** ^1^Department of Hematology, The Affiliated Tai'an City Central Hospital of Qingdao University, Tai'an, Shandong, China; ^2^Department of Gastroenterology, The Affiliated Tai'an City Central Hospital of Qingdao University, Tai'an, Shandong, China; ^3^Department of Cardiology, The Affiliated Tai'an City Central Hospital of Qingdao University, Tai'an, Shandong, China; ^4^Department of Clinical Laboratory, The Affiliated Tai'an City Central Hospital of Qingdao University, Tai'an, Shandong, China

**Keywords:** fungal infection, diabetes, mucormycosis, intestinal infection, *Lichtheimia ramosa*

## Abstract

Mucormycosis is an aggressive fungal disease. Gastrointestinal mucormycosis is rare, but its clinical symptoms lack specificity and mortality is high. Here, we report a case of intestinal mucormycosis caused by *Lichtheimia ramosa* in a 65-year-old woman with diabetes mellitus. The patient exhibited extensive mucosal tissue damage in the colon, with broad, undivided filamentous fungal hyphae present in the intestinal tissue. Therefore, the patient was suspected to have a filamentous fungal infection. Colonic tissue samples were obtained for fungal culture, and the fungus was identified as *L. ramosa* based on morphology and DNA sequencing. This case highlights the importance of pathologists and microbiologists in identifying pathogenic fungi and the significance of screening for mucormycosis in high-risk patient populations.

## 1 Introduction

Mucormycosis is a life-threatening, invasive fungal infection caused by fungi from the subphylum *Mucoromycotina*, order *Mucorales*, which primarily affects immunocompromised patients (1). These pathogens can infect any organ system, including the head, central nervous system, neck, skin, nasal cavity, lungs, and gastrointestinal tract (GI) (2). The most common sites of invasion are the rhinocerebral and pulmonary regions, with GI infections being the rarest (3).

GI mucormycosis accounts for only 5–15% of all mucormycosis cases but has one of the highest mortality rates among its clinical presentations (4). Common risk factors for GI mucormycosis in adults include diabetes, stem cell or solid organ transplantation, corticosteroid therapy, malnutrition, intestinal digestive surgery, and hemodialysis/peritoneal dialysis (4, 5). This infection most frequently occurs in the stomach, followed by the colon, small intestine, and esophagus, which are less common (6). *Lichtheimia ramosa*, previously known as *Absidia ramose* and a member of the saprotrophic zygomycetous fungi, is an uncommon pathogen within the order Mucorales (7). Herein, we present a rare case of invasive intestinal mycosis caused by *L. ramosa.*

## 2 Case presentation

On March 7th, 2022, a 65-year-old woman was admitted to the hospital with nausea, vomiting, and diarrhea. Initial treatment included antibiotic therapy and fluid replenishment to maintain electrolyte balance. On March 9th, she was transferred to our hospital for further management due to lip edema, gingival congestion, and dyspnea. During this period, she experienced over ten episodes of watery diarrhea accompanied by periumbilical pain, although she did not have a fever. Her medical history included diabetes, hypertension, and cerebral infarction, with no reported infectious diseases or exposure to similar illnesses. Despite her diabetes, she was not on antidiabetic medications; however, she continued her antihypertensive treatment. Physical examination revealed a body temperature of 36.6°C, blood pressure of 153/94 mm Hg, lip swelling, gingival congestion, rough breathing sounds, and audible wheezing.

**Figure 1:**
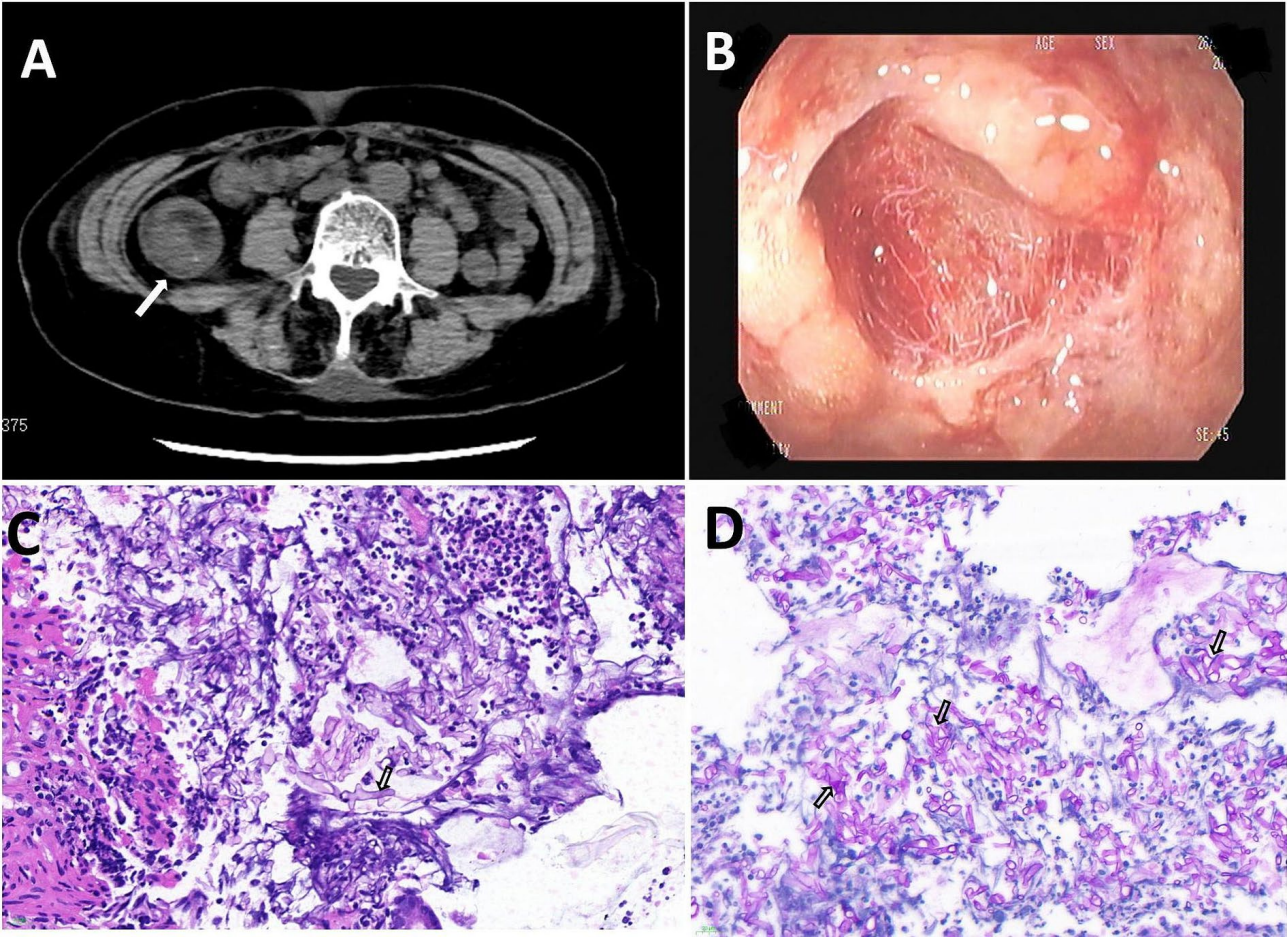
**(A)** Computed tomography (CT) revealed bowel wall thickening. **(B)** Gastroscopy revealed severe erosions, edema, and scattered superficial ulcers. **(C,D)** A broad, aseptate hyphae filamentous fungi was found in the intestinal tissue (Magnification: ×400). The tissue specimens were stained with Hematoxylin and Eosin staining **(C)**, periodic acid Schiff **(D)**.

Subsequent laboratory investigations after admission (March 9th) revealed a leukocyte count of 11.96 × 10^9^/L (normal: 3.5–9.5 × 10^9^/L), a neutrophil count of 11.27 × 10^9^/L (normal: 1.8–6.3 × 10^9^/L), and a lymphocyte count of 0.41 × 10^9^/L (normal: 1.1–3.2 × 10^9^/L), with a lymphocyte percentage of 3.4% (normal: 20–50%). Procalcitonin level was measured at 0.18 ng/mL (normal: 0–0.5 ng/mL), interleukin-6 at 67.97 pg./mL (normal: 0–5.4 pg./mL), fasting blood glucose at 12.31 mmol/L (normal 3.9–6.1 mmol/L), alanine transaminase at 527 U/L (normal: 7–40 U/L), aspartate transaminase at 556.3 U/L (normal: 13–35 U/L), and creatinine at 378 μmol/L (normal: 41–81 μmmol/L). Microbiological tests for *Clostridium difficile* toxin, *Salmonella* and *Shigella* spp., *Aspergillus* antigen (GM test), and fungal 1,3-*β*-D-glucan (G test) were all negative. A lymphocyte subpopulation test conducted 7 days after admission revealed a CD3+ T-cell count of 572/μL (normal: 955–2,860/μL), a CD4+ T-cell count of 304/μL (normal: 550–1,440/μL), a CD8+ T-cell count of 176/μL (normal: 320–1,250/μL), and a natural killer cell count of 34/μL (normal: 150–1,100/μL). Abdominal computed tomography revealed multiple hypodense foci in the liver, localized wall thickening in the lower intestine, and enlarged mesenteric lymph nodes (Figure 1A).

The patient presented with bloody stools containing red mucosal tissue, indicative of intestinal mucosal damage. On March 14th, a colonoscopy revealed hyperemia, edema, erosion, and scattered superficial ulcers in the ileocecal region and throughout the colon, covered with a large amount of white material (Figure 1B) resistant to rinsing with dilute amphotericin B.

**Figure 2:**
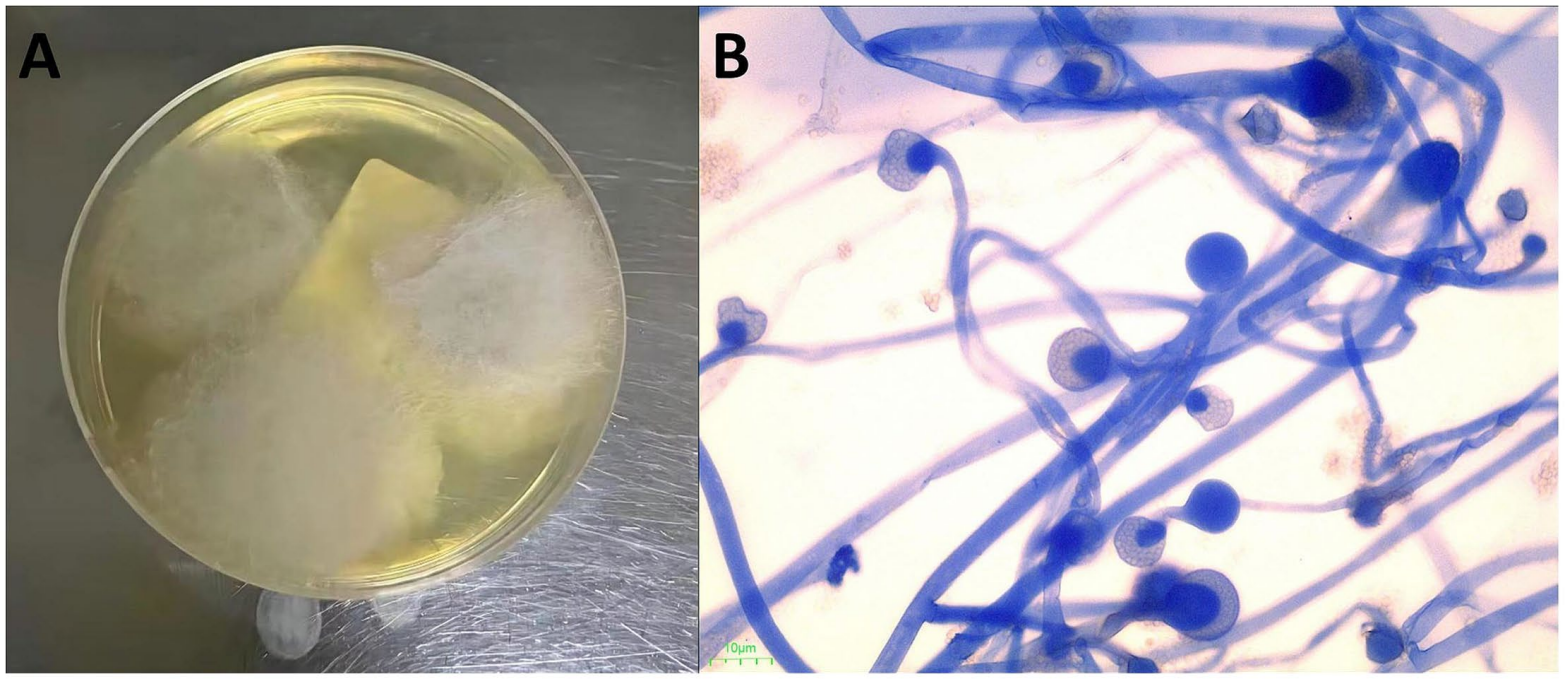
**(A)** White cottony growth of *L. ramosa* on the Sabouraud dextrose agar (SDA). **(B)** A lactophenol cotton blue (LCB) preparation showing pear-shaped sporangia and prominent conical columella.

Microscopic examination of a colonic biopsy specimen showed severe chronic inflammation, acute active inflammation, cryptic inflammation, crypt abscesses, inflammatory exudation, and necrosis. Broad and undivided filamentous fungal hyphae were detected in the intestinal tissue using hematoxylin and eosin (HE) (Figure 1C) and Schiff periodate (PAS) staining (Figure 1D). Colon tissue samples cultured on Sabouraud agar medium exhibited colonies with grayish-white hyphae, a flat surface, and a velvety texture, rapidly spreading across the entire plate (Figure 2A). Further microscopic examination of lactophenol cotton blue stain revealed broad, aseptate branching hyphae. The sporangium stems, originating from creeping hyphae, were pear-shaped and had prominent conical columella (Figure 2B). DNA was extracted from the culture and subjected to sequencing with a fungal universal 18S rRNA primer pair (V4 regions: 3NDF, 5′-GGCAAGTCTGGTGCCAG-3′; and V4eukR2R, 3′-ACGGTATCTRATCRTCTTCG-5′). The colony was identified as *L. ramosa* on March 23th.

**Figure 3:**
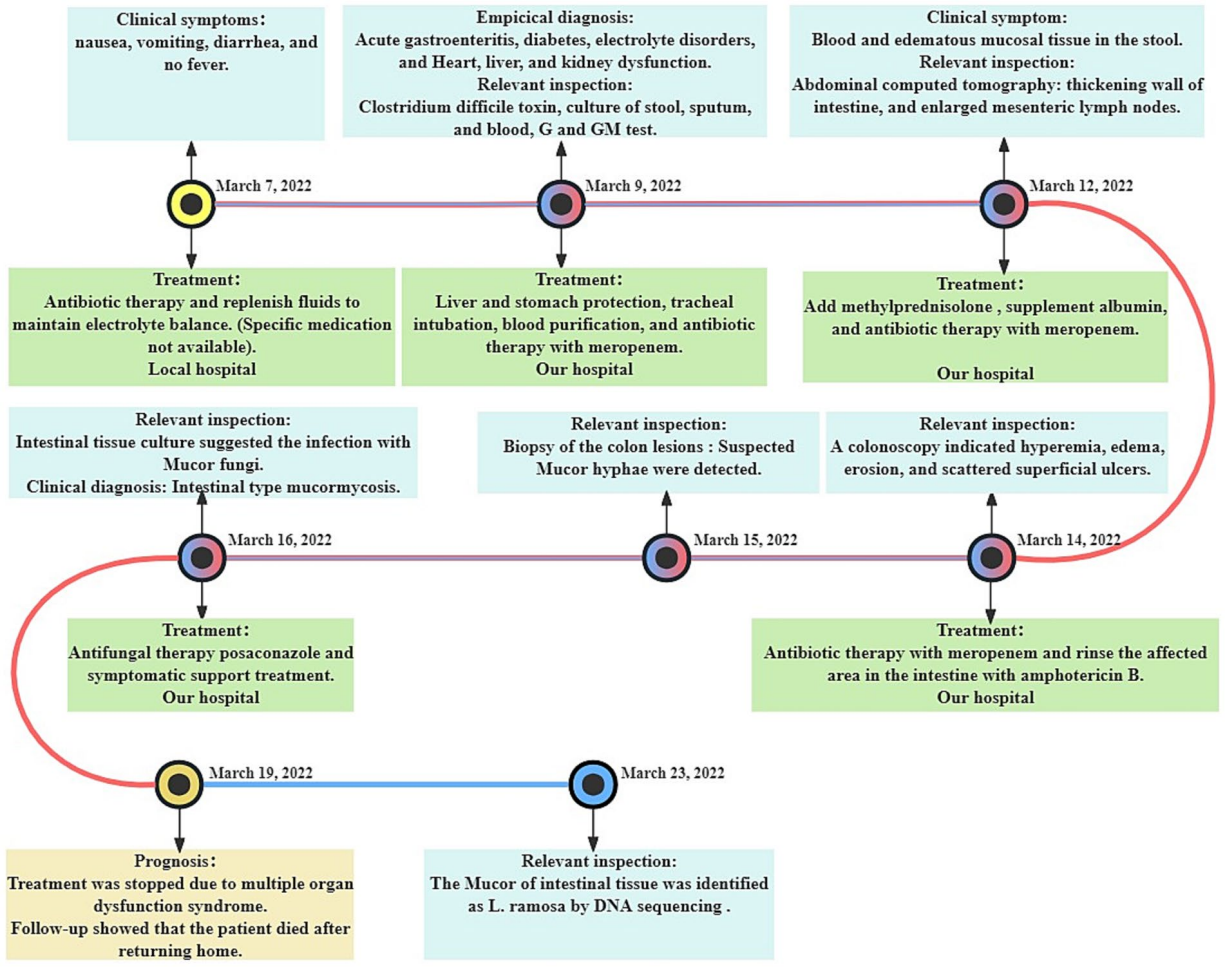
Timeline of the disease course.

After admission, the patient was treated with meropenem, cefoperazone/sulbactam, and hormone therapy. Based on intestinal histopathologic and tissue culture results, a *mucorales* infection was suspected, and the patient was diagnosed with intestinal-type mucormycosis on March 16th. Posaconazole was administered immediately for antifungal treatment. Despite prompt intervention, the disease progressed rapidly. Regrettably, the patient’s family decided to discontinue treatment on March 19th, 2022, due to the presence of multiple organ dysfunction syndrome. Subsequent follow-up indicated that the patient passed away post-discharge (Figure 3).

## 3 Discussion

Gastrointestinal mucormycosis caused by *L. ramosa* is a rare opportunistic infection (8). The increasing use of glucocorticoids and immunosuppressants annually is also contributing to the rising incidence of mucormycosis. Diabetes has been a leading factor in mucormycosis and one of the important causes of fungal infections in humans (9). In China, poorly controlled diabetes has been identified as the most important risk factor for mucormycosis (10), similar to that in India, South America, the Middle East, and Africa. Mucormycosis commonly affects diabetic patients with uncontrolled blood sugar levels, predominantly causing rhino-orbito-cerebral infections due to *Rhizopus* species. However, gastrointestinal manifestations are rare, and infections caused by *L. ramosa* at the intestinal site are even more uncommon. Here, we present a fatal case of invasive intestinal infection with *L. ramosa* in a patient with poorly controlled diabetes mellitus.

The most frequently reported manifestations in patients of GI mucormycosis were abdominal pain, distention and fever, often depend on the area affected of the gastrointestinal tract. With intestinal mucormycosis, almost all patients have been reported to experience abdominal pain and fever (7, 11–13). Our patient has abdominal pain and diarrhea, but no fever. There are patients who were admitted for other reasons, such as a patient who was admitted due to shock, high fever, hypoxic respiratory failure and generalized symptoms of shaking and then found to have mucormycosis in the intestines (14). *L. ramose* is an opportunistic fungal pathogen that may result in a rare but serious mucormycosis infection. Mucormycosis caused by *L. ramose* infection was mainly found in the lungs, rhino-orbito-cerebral and skin (8, 15–17), and the intestinal mucormycosis caused by *L. ramose* is rare. There was only one report of a 53-year-old female patient with intestinal mycosis due to perforation of the transverse colon following antibacterial chemotherapy after esophageal cancer surgery, resulting in co-infection with *L. ramose* and *Aspergillus calidoustus* (7). The exact mechanism by which *L. ramosa* invades the intestine remains unclear, and may be related to the ingestion of spore-infected food.

GI mucormycosis is a highly invasive, virulent fungal infection. Early endoscopic biopsy is key to diagnosis. At present, the gold standard for the diagnosis of GI mucormycosis is tissue histopathology, which can diagnose 70% of cases. Culture and mucorales polymerase chain reaction (PCR) of tissue adds to the diagnostic yield (18). Semi-nested PCR for 18S rRNA gene and quantitative real-time PCR assays targeting the 28S rRNA gene are also molecular methods for early diagnosis of *Mucorales* (6). Mucormycosis lacks the cell wall components that can be detected on serum tests such as the 1,3-beta-D-glucan (BG) assay, which led to a negative test for the BG test. Therefore, clinicians should exercise caution and not exclude *Mucorales* infections solely based on G and GM test results. Instead, microbiological and histopathological examinations should be actively conducted in high-risk patients. Staining techniques such as PAS or silver hexamide staining of the tissue are recommended to enhance visualization of the fungal composition. In our patient, filamentous fungi were clearly detected by HE and PAS staining of intestinal mucosal tissue. Many patients are ineligible for surgery or endoscopic biopsy because of their poor physical condition, with most cases identified during autopsy. Even with the fungal culture of the intestinal tissue, there’s still no fungal growth in culture in more than 50% of cases (3, 19). Gastrointestinal tissue is not sterile tissue, and cultures will not be able to differentiate between harmless contamination and invasive infection. Therefore, the proportion of patients with confirmed premortal diagnoses of *Mucorales* spp. by tissue culture is quite small. In our case, the fungus was successfully cultured from the colon biopsy tissue and identified as *L. ramosa* via DNA sequencing using a fungal universal 18S rRNA primer pair.

The prognosis after the onset of gastrointestinal tract infection with *Mucorales* is poor, with a high mortality rate (20). Early surgical intervention is crucial in treating mucormycosis, including local debridement and the removal of infected tissues or organs when conditions permit. Additionally, timely and effective systemic antifungal therapy for mucormycosis is necessary after a definitive diagnosis. According to the literature, the empirical antifungal treatments for *L. ramosa* are amphotericin B and posaconazole. Amphotericin B is the most active drug against *Lichtheimia* spp. (21). In a case report, intestinal mycosis caused by co-infection with *L. ramosa* and *Aspergillus calidoustus* was successfully treated with liposomal amphotericin B after an emergency subtotal colectomy (7). However, amphotericin B has adverse effects, specifically severe nephrotoxicity. Furthermore, although amphotericin B liposomes can reduce nephrotoxicity, they should be used with caution in patients with renal impairment. Posaconazole is a recommended therapy that can serve as a complement or substitute for amphotericin B therapy (16). However, the efficacy of posaconazole remains controversial (22). In this study, the intestinal lesions of the patient were extensive and her physical condition was poor; therefore, surgery could not be performed. Due to compromised kidney function, the patient was treated with posaconazole instead of amphotericin B. Unfortunately, the patient discontinued treatment 10 days after admission and subsequently passed away at home. Hence, timely diagnosis and treatment are required to increase the chances of returning to health.

## 4 Conclusion

Intestinal mycosis can be difficult to diagnose and manage, owing to the various forms of the disease and the need to obtain and analyze tissue specimens at an early stage at an appropriate facility. The dramatic increase in *Mucorales* fungal infections demands more rapid and accurate diagnostic methods to identify such pathogens. We should actively control the basic diseases of susceptible people, and patients with high-risk factors should avoid contact with *Mucorales*-contaminated items and take anti-mold measures. Clinicians should remain highly suspicious of emerging fungal infections in high-risk patients, such as those with diabetes.

## Data Availability

The original contributions presented in the study are included in the article/supplementary material, further inquiries can be directed to the corresponding author.
